# The association of Chinese and American antenatal care utilization indices with birth outcomes

**DOI:** 10.3389/fpubh.2024.1420943

**Published:** 2024-08-07

**Authors:** Haibo Zhou, Yi Yang, Peihan Chi, Haoyue Cheng, Xialidan Alifu, Yiwen Qiu, Ye Huang, Libi Zhang, Diliyaer Ainiwan, Yan Zhuang, Hui Liu, Zhi Chen, Yunxian Yu

**Affiliations:** ^1^Department of Public Health and Department of Anesthesiology, Second Affiliated Hospital of Zhejiang University School of Medicine, Hangzhou, China; ^2^Department of Epidemiology and Health Statistics, School of Public Health, School of Medicine, Zhejiang University, Hangzhou, China; ^3^Department of Science and Education of the Fourth Affiliated Hospital, and Center for Health Policy Studies, School of Public Health, Zhejiang University School of Medicine, Hangzhou, China; ^4^Clinical Research Center, Sir Run Run Shaw Hospital, School of Medicine, Zhejiang University, Hangzhou, China; ^5^State Key Laboratory for Diagnosis and Treatment of Infectious Diseases, National Clinical Research Center for Infectious Diseases, Collaborative Innovation Center for Diagnosis and Treatment of Infectious Diseases, The First Affiliated Hospital, College of Medicine, Zhejiang University, Hangzhou, China

**Keywords:** prenatal care, birth weight, index, SGA, preterm birth, high-risk pregnancy

## Abstract

**Objective:**

Few comparisons have been implemented between different prenatal care utilization indices and their effects on adverse outcomes. This study investigated the appropriateness of Chinese antenatal care (ANC) regulations and compared Chinese and American adequacy of prenatal care utilization (APNCU) scores.

**Methods:**

From 2010 to 2022, the medical records of 60,114 pregnant women were collected from the electronic medical record system (EMRS) in Zhoushan, China. ANC utilization was measured using the APNCU score and five times antenatal care (ANC5). Birth weight outcomes, including small for gestational age (SGA) and large for gestational age (LGA), low birth weight (LBW), macrosomia, birth weight, and preterm birth (PTB), were utilized as outcomes. Multinomial, linear, and logistic regression were used to analyze the association of ANC5 and APNCU with outcomes, respectively. Crossover analysis was implemented to compare the interaction between ANC5 and APNCU on the outcomes.

**Results:**

Women who received inadequate prenatal care had increased odds for PTB (ANC5: odds ratio (OR) = 1.12, 95% confidence interval (95%CI) = 1.03–1.21; APNCU: OR = 1.18, 95%CI: 1.07–1.29), delivering SGA infants (ANC5: OR = 1.13, 95%CI = 1.07–1.21; APNCU: OR = 1.11, 95%CI = 1.03–1.20). Crossover analysis revealed that inadequate prenatal care in APNCU only was significantly associated with an increased risk of PTB (OR = 1.48, 95%CI: 1.26–1.73).

**Conclusion:**

Women with inadequate prenatal care in ANC5 or APNCU were more likely to suffer from adverse birth outcomes, including PTB, birth weight loss, SGA, and LBW. It indicated that adequate prenatal care is necessary for pregnant women. However, there were interactions between ANC5 and APNCU on PTB, with inadequate prenatal care use by APNCU showing the highest risk of PTB. This indicates that APNCU would be a better tool for evaluating prenatal care use.

## 1 Introduction

Prenatal care is a common health intervention that may lower the incidence of perinatal morbidity and death by treating medical disorders, detecting and minimizing possible hazards, and assisting women in avoiding unhealthy life behaviors that contribute to adverse outcomes (1), such as small for gestational age (SGA) (1), preterm birth (PTB) (2), and low birth weight (LBW) (3). The effect of these outcomes was evaluated to be associated with distress syndrome, infections, and developmental delays, which can extend into adulthood, leading to chronic conditions such as cardiovascular disease and diabetes (4).

Measurements of prenatal care have evolved from pure prenatal care frequency (5) to indices. The first prenatal care index was proposed by Kessner (6) and is called the Kessner Index. After that, many other indices were developed. These indices have been employed in studies assessing prenatal care and birth outcomes to evaluate the appropriateness of prenatal care (7). The Adequacy of Prenatal Care Utilization (APNCU) score is an improvement on the Institute of Medicine’s Kessner Index, which incorporates the trimester of prenatal care commencement and the number of prenatal visits (8). The APNCU index is commonly used for accurate and complete prenatal care measurement (9). Previous studies have shown that APNCU was associated with SGA (1), PTB (2), and LBW (3).

However, the requirements for prenatal care visits vary in different countries (10). In Canada, the Society of Obstetricians and Gynaecologists of Canada (SOGC) mentions that women in developed countries typically have 7 to 11 regular prenatal visits throughout each pregnancy (11). The World Health Organization recommended that women complete at least four times of prenatal care in 2002 and adjusted the frequency to eight times in 2016 (12). In China, although most hospitals adopt suggestions from the American College of Obstetricians and Gynecologists (ACOG), which requires women to attend antenatal care (ANC) once in different intervals in three periods of gestation, the government document National Basic Public Health Service Standard (NBPHS) requires that pregnant women pay at least one visit during five periods in pregnancy. The five corresponding gestational age periods were ≤ 13th, 16 ~ 20th week, 21 ~ 24th, 28 ~ 36th, and 37 ~ 40th week of gestation. For convenience, this five-time-specific ANC was modified to ANC5, which defines pregnant women who obtain at least five times ANC during pregnancy, regardless of the timing period, as having received adequate prenatal care (13, 14). Therefore, it is necessary to estimate the association between ANC5 and adverse birth outcomes.

Furthermore, to comprehensively assess the impact of prenatal care, it is crucial to consider various sociodemographic and medical factors. This study incorporates variables such as age, education level (15), BMI (16), gestational age at the first visit, maternal high-risk pregnancy status, smoking and alcohol consumption during or before pregnancy (17), and parity, which has been reported to be related to maternal adverse outcomes (18). Specifically, high-risk pregnancy (19) status is a critical factor as it may confound the association between prenatal care utilization and birth outcomes. Women with high-risk pregnancies often require more intensive prenatal care, and their adverse outcomes might reflect their underlying risk rather than the adequacy of care received. These variables are selected based on the Andersen Behavioral Model (20), which provides a framework for understanding health service utilization. According to this model, health behaviors and outcomes are influenced by predisposing factors (e.g., education level, pre-pregnancy behaviors, and parity), enabling factors (e.g., BMI and gestational age of the first visit), and need factors (e.g., high-risk pregnancy status). By incorporating these variables, the study aims to provide a more thorough assessment of prenatal care adequacy and its impact on birth outcomes.

In addition, a few studies have been implemented to compare different indices, such as Gindex and Kessner index (21, 22) on different birth outcomes. However, few studies have evaluated which ANC utilization indices, such as APNCU and ANC5, are more suitable for clinical practice. This could be due to the complexity of accurately measuring prenatal care adequacy and the variations in healthcare systems and guidelines across different regions. Additionally, the lack of standardized criteria for evaluating prenatal care indices and the challenges in collecting comprehensive and consistent data may contribute to the limited number of comparative studies. It also needs to be validated whether the Chinese population should use the ACOG prenatal care program or if there is a better way to evaluate prenatal care utilization adequacy. Therefore, this study also aims to compare the association between different prenatal care utilization indices and adverse birth outcomes.

## 2 Methods

### 2.1 Data source

The data were retrieved from the electronic medical record system (EMRS) in Zhoushan City, Zhejiang Province, China. The EMRS, a municipal system built in Zhoushan in 2001, had prenatal health and birth registration data. The EMRS only included the data from Zhoushan Maternal and Child Care Hospital from 2001 to 2009. Starting in 2010, the EMRS covered maternal and pediatric medical information from the whole city.

The prenatal health dataset was used to extract maternal data about sociodemographic information (such as maternal age, educational attainment, parity, last menstrual period, and follow-up date) and health-related features (such as maternal height, weight, systolic and diastolic blood pressure during pregnancy, and liver and kidney diseases). Birth data (such as neonatal gender, birth weight, and gestational age) was also collected. Both databases were connected by a unique personal identification number. The study protocol was approved by the institutional review board of Zhejiang University School of Medicine.

### 2.2 Study populations

Considering the representation of the population, medical records that included all the people in Zhoushan City after 2010 were included. In this investigation, women who visited hospitals between 2010 and 2022 were considered. Age between 18 and 45 years, first visit after the year 2010, and gestational age at delivery between 28 and 42 weeks were the inclusion criteria for this study. The women who met any one of the following conditions were excluded: (1) multiple births and (2) birth weights of more than 5,000 g or less than 1,000 g. The detailed data flow is presented in Supplementary Figure S1.

### 2.3 The definition of prenatal care utilization (APNCU and ANC5)

#### 2.3.1 APNCU

A prenatal care visit is a medical appointment that pregnant women attend during pregnancy to receive healthcare services from medical professionals. If many visits were identified at the exact location on the same day, one visit would be recorded. Prenatal care usage was evaluated by APNCU and ANC5. Original APNCU is divided into four categories and calculated based on two components: the month prenatal care began and the number of visits from the first prenatal care to delivery (8). Inadequate ANC utilization is defined as beginning prenatal care after the fourth month of pregnancy or obtaining less than 50% of the needed prenatal care visits based on the ACOG schedule (0–28th week: 1 visit/4 weeks; 29–36th week: 1 visit/2 weeks; 37–42th week: 1 visit/week). Intermediate ANC utilization is initiated before or equal to the fourth month of pregnancy, with 50–79% of routine visits. Adequate ANC utilization is undertaken by the fourth month of pregnancy, with 80–109% of planned visits. Adequate plus ANC utilization is initiated by the fourth month of pregnancy with 110% or more of expected visits (8).

#### 2.3.2 ANC5

ANC5 was divided into adequate or inadequate. Adequate group means that women obtained at least one prenatal care visit in each time period: ≤13th, 14 ~ 20th, 21 ~ 27th, 28 ~ 36th, and ≥ 37th week of gestation recommended by NBPHS, respectively. Inadequate means that women only completed prenatal care visits in no more than four different periods mentioned above. Additionally, considering that some women might miss the prenatal care visit after the 37th week due to premature delivery from the 29th to the 37th week, these women would be categorized into adequate groups if they completed prenatal care visits in four different periods. Women delivering at the 28th week would also be categorized into adequate groups if they completed prenatal care visits in three different periods.

To compare the association between APNCU and ANC5, APNCU was transformed into a binary variable: adequate plus and adequate were merged into adequate, while intermediate and inadequate were merged into inadequate. Additionally, prenatal care frequency was included and divided into quartiles to facilitate the comparison of their differences. For ease of reading, the frequency in parentheses indicates the range of values within each quartile category: Q1 (1 ~) indicates a frequency from 1 to 10, Q2 (11 ~) indicates a frequency from 11 to 12, Q3 (13 ~) indicates a frequency from 13 to 14, and Q4 (14 ~) indicates a frequency from 14 onwards.

### 2.4 Outcomes

The primary outcomes are PTB, SGA, and LGA. PTB was defined as babies born before 37 weeks of gestation. The birth weight with less than the 10th or greater than the 90th percentile for gestational age was defined as SGA and LGA, respectively. An infant with a birth weight between the 10th and 90th percentile was defined as appropriate for gestational age (AGA). Secondary outcomes are birth weight (g), LBW, and fetal macrosomia (FM). LBW and FM were determined if the birth weight was less than 2,500 g or greater than 4,000 g, respectively.

### 2.5 Covariates

The potential confounding factors included maternal educational level, risk of pregnancy (low and high), body mass index (BMI), gestational age of the first visit, gestational age of delivery, calendar year of the first visit, alcohol drinking (yes and no) or cigarette smoking (yes and no) before or during pregnancy, and parity (primipara and multipara). Educational level was separated into three tiers (middle school and less, high school and college, and more). Risk pregnancy includes, but is not limited to, situations before or during pregnancy: obstetric history, such as history of cesarean delivery, history of uterine rupture, and history of multiple induced abortions; pregnancy complications, such as excessive amniotic fluid, premature rupture of membranes, fetal growth restriction, and severe pre-eclampsia; and maternal medical conditions, such as epilepsy requiring pharmacological control, unstable thyroid disease, severe anemia, and pulmonary hypertension. It was determined by health professionals. A detailed high risk is defined by the items in Supplementary Table S1. It was divided into high-risk and low-risk groups. High risk means if any of the conditions in Supplementary Table S1 occurred during pregnancy and low risk means none of the situations occurred. The BMI was calculated by weight in kilograms divided by the square of standing height in meters and then categorized into four groups (underweight: <18.5 kg/m^2^; normal weight: 18.5 ~ 23.9 kg/m^2^; overweight: 24 ~ 27.9 kg/m^2^; and obesity: ≥28 kg/m^2^). The gestational week that women first visited the hospital during the current pregnancy to receive prenatal care was defined as the gestational age of the first visit. The gestational week was defined by the birth date subtracted from the first day of the last normal menstrual cycle and/or the date established by an ultrasound.

### 2.6 Sensitivity analysis

Since the time for women to participate in ANC is different, some women with low ANC utilization may start the physical examination in the third trimester of pregnancy. Therefore, women with pre-pregnancy BMI were utilized. We compare the association between prenatal care utilization and adverse outcomes mentioned above with pre-pregnancy BMI and BMI at the first visit, respectively. Considering the sample size reduced drastically, we divided the prenatal care frequency into quartiles within the subgroup analysis. Furthermore, risk pregnancy was believed to be a potential leading bias in the analysis. Therefore, a stratification analysis by risk pregnancy was implemented.

### 2.7 Statistical analyses

The random forest was used to impute missing values for variables with fewer than 10% of missing data. Continuous variables were presented as means and standard deviations (mean ± SD). For categorical data, frequencies and percentages (%) were presented. The analysis of variance (ANOVA) and chi-square test were used to compare continuous and categorical data, respectively. The association between prenatal care utilization and adverse pregnancy outcomes was visualized through restricted cubic spline (RCS). Due to the non-linear relationship between prenatal care utilization and adverse pregnancy outcomes, the total number of prenatal care visits was treated as quartile (Q1 ~ Q4). Multiple linear regression was used to analyze the association between prenatal care utilization (including the total number of prenatal care visits, APNCU, and ANC5) and birth weight (continuous variable). The model was adjusted for the following potential confounders: maternal age, educational level, BMI, risk diagnosis, parity, calendar year of the first visit, alcohol or cigarette consumption, gestational age of the first visit, and gestational age of delivery. Multiple logistic regression was used to assess the association between prenatal care utilization and PTB, after adjustment for covariates mentioned above, except the gestational age of delivery. Multinomial logistic regression was used to determine the relationship between prenatal care utilization and categorical outcomes of birth weight (SGA, normal, and LGA; LBW, normal, and macrosomia). The crossover analysis was used to explore the interaction between ANC5 and APNCU on outcomes. Covariates were the same as those in the previous models of corresponding outcomes. All statistical analyses were performed using R (version 4.3.1). The statistical significance was considered as a *p*-value of <0.05.

## 3 Results

### 3.1 Distribution of maternal characteristics between prenatal care utilization

Table 1 presents the distribution of maternal characteristics between prenatal care utilization groups. This study included 60,114 pregnant women. The average age and gestational age of delivery were 28.4 ± 4.22 years and 39.09 ± 1.49 weeks, respectively. Most women took their first visit of prenatal care at 12.16 ± 3.33 weeks of gestation. More than half of women received college or higher education (55.2%). Almost 18% of pregnant women were overweight or obese. Most women experienced high-risk pregnancies (60.1%) and primipara (68.7%). Overall, the number of newborns was similar in each calendar year. A few women consumed alcohol or cigarettes (1.3%) before or during pregnancy. Most of the women received adequate prenatal care, despite being estimated by ANC5 (69.6%) or APNCU (82.1%).

**Table 1:** Distribution of socio-economic characteristics.

Variable	Overall (*n* = 60,114)
	Mean ± SD
Age	28.40 ± 4.22
Gestational age of delivery	39.09 ± 1.49
Gestational age of the first visit	12.16 ± 3.33
	*N* (%)
**Education**	
Middle school or less	15,497 (25.80)
High school	11,457 (19.10)
College or more	33,160 (55.20)
**BMI at the first visit**	
Underweight	8,834 (14.70)
Normal	40,526 (67.40)
Overweight	8,495 (14.10)
Obesity	2,259 (3.80)
**Risk pregnancy**	
Low risk	23,977 (39.90)
High risk	36,137 (60.10)
**Parity**	
Primipara	41,315 (68.70)
Multipara	18,799 (31.30)
**Calendar year of the first visit**	
2010 ~	12,533 (20.80)
2013 ~	16,981 (28.20)
2016 ~	15,453 (25.70)
2019 ~	15,147 (25.20)
**Alcohol or cigarette**	
No	59,303 (98.70)
Yes	811 (1.30)
**Original APNCU**	
Adequate plus	10,080 (16.80)
Adequate	39,272 (65.30)
Intermediate	7,211 (12.00)
Inadequate	3,551 (5.90)
**ANC5**	
Adequate	41,813 (69.60)
Inadequate	18,301 (30.40)
**APNCU**	
Adequate	49,352 (82.10)
Inadequate	10,762 (17.90)

### 3.2 Association between prenatal care utilization and birth outcome

Table 2 shows the association between prenatal care utilization and PTB. Inadequate ANC5 (OR = 1.12, 95%CI:1.03–1.21) and APNCU (OR = 1.18, 95%CI: 1.07–1.29) were associated with PTB. Additionally, it could be inferred that an increase in prenatal care was negatively related to PTB (Q4: OR = 0.40, 95%CI: 0.36–0.45).

**Table 2:** Association between prenatal care utilization and PTB.

Variable	PTB		Model 1^a^	Model 2^b^
	No	Yes	OR (95%CI)	
**Prenatal care visit**				
Q1 (1 ~)	19,727 (34.96)	2,505 (68.09)	Ref.	Ref.
Q2 (11 ~)	10,596 (18.78)	275 (7.47)	0.20 (0.18, 0.23)^‡^	0.19 (0.17, 0.22)^‡^
Q3 (12 ~)	19,338 (34.27)	517 (14.05)	0.21 (0.19, 0.23)^‡^	0.19 (0.18, 0.21)^‡^
Q4 (14 ~)	6,774 (12.00)	382 (10.38)	0.44 (0.40, 0.50)^‡^	0.40 (0.36, 0.45)^‡^
**ANC5**				
Adequate	39,406 (69.83)	2,407 (65.43)	Ref.	Ref.
Inadequate	17,029 (30.17)	1,272 (34.57)	1.22 (1.14, 1.31)^‡^	1.12 (1.03, 1.21)^†^
**APNCU**				
Adequate	46,457 (82.32)	2,895 (78.69)	Ref.	Ref.
Inadequate	9,978 (17.68)	784 (21.31)	1.26 (1.16, 1.37)^‡^	1.18 (1.07, 1.29)^‡^

^†^ *p* < 0.01; ^‡^ *p* < 0.001. PTB, preterm birth. ^a^ model was crude; ^b^ model was additionally adjusted with age, education, BMI, risk pregnancy, parity, calendar year of the first visit, alcohol or cigarette, and gestational age of the first visit.

Table 3 displays the relationship between prenatal care utilization and SGA or LGA. Inadequate prenatal care utilization measured in ANC5 (OR = 1.13, 95%CI: 1.07–1.21) and APNCU (OR = 1.11, 95%CI: 1.03–1.20) were associated with an increased risk of SGA. Increasing prenatal care utilization was associated with a lower risk of SGA (Q4: OR = 0.84, 95%CI: 0.77–0.92) compared to Q1. Moreover, an elevation in prenatal care utilization was associated with an increased risk of LGA (Q4, OR = 1.23, 95%CI: 1.12–1.35). Supplementary Tables S3A,B shows the association between prenatal care visits and birth weight and LBW/FM, respectively. Similar to previous results, inadequate prenatal care in both ANC5 and APNCU was associated with weight loss [ANC5: β (se) = −14.21 (3.87); APNCU: β (se) = −26.68 (4.57)]. However, inadequate ANC5 and APNCU were not associated with LBW.

**Table 3:** Association between prenatal care utilization and SGA and LGA.

Variable	AGA	SGA			LGA		
	*N* (%)	*N* (%)	Model 1^a^	Model 2^b^	*N* (%)	Model 1^a^	Model 2^b^
			OR (95%CI)			OR (95%CI)	
**Prenatal care visit**							
Q1 (1 ~)	17,288 (36.83)	2,692 (38.86)	Ref.	Ref.	2,252 (36.05)	Ref.	Ref.
Q2 (11 ~)	8,414 (17.93)	1,301 (18.78)	0.99 (0.92, 1.07)	0.96 (0.89, 1.03)	1,156 (18.50)	1.05 (0.98, 1.14)	1.11 (1.03, 1.20)^†^
Q3 (12 ~)	15,696 (33.44)	2,160 (31.18)	0.88 (0.83, 0.94)^‡^	0.83 (0.78, 0.88)^‡^	1999 (32.00)	0.98 (0.92, 1.04)	1.05 (0.98, 1.13)
Q4 (14 ~)	5,541 (11.80)	775 (11.19)	0.90 (0.82, 0.98)^*^	0.84 (0.77, 0.92)^‡^	840 (13.45)	1.16 (1.07, 1.27)^‡^	1.23 (1.12, 1.35)^‡^
**ANC5**							
Adequate	32,818 (69.92)	4,698 (67.81)	Ref.	Ref.	4,297 (68.79)	Ref.	Ref.
Inadequate	14,121 (30.08)	2,230 (32.19)	1.10 (1.05, 1.16)^‡^	1.13 (1.07, 1.21)^‡^	1950 (31.21)	1.05 (1.00, 1.12)	1.03 (0.96, 1.09)
**APNCU**							
Adequate	38,634 (82.31)	5,615 (81.05)	Ref.	Ref.	5,103 (81.69)	Ref.	Ref.
Inadequate	8,305 (17.69)	1,313 (18.95)	1.09 (1.02, 1.16)^*^	1.11 (1.03, 1.20)^†^	1,144 (18.31)	1.04 (0.97, 1.12)	1.02 (0.94, 1.10)

^*^p < 0.05; ^†^*p* < 0.01; ^‡^*p* < 0.001. AGA, appropriate for gestational age; SGA, small for gestational age; LGA, large for gestational age. ^a^ model was crude; ^b^ model was additionally adjusted with age, education, BMI, risk pregnancy, parity, calendar year of the first visit, alcohol or cigarette and gestational age of the first visit.

### 3.3 Crossover analysis of ANC5 and APNCU on birth outcome

The crossover analysis of ANC5 and APNCU on preterm showed that compared to women with both adequate ANC5 and APNCU, only inadequacy in ANC5 (OR = 1.15, 95%CI: 1.05–1.26) or APNCU (OR = 1.48, 95%CI: 1.26–1.73) was both associated with an increased risk of PTB (Table 4). However, a joint effect was not detected.

**Table 4:** Crossover analysis of APNCU on PTB.

ANC5	APNCU	PTB		Model 1^a^	Model 2^b^
		No	Yes	OR (95%CI)	
Adequate	Adequate	37,344 (66.17)	2,233 (60.70)	Ref.	Ref.
Adequate	Inadequate	2062 (3.65)	174 (4.73)	1.41 (1.20, 1.65)^‡^	1.48 (1.26, 1.73)^‡^
Inadequate	Adequate	9,113 (16.15)	662 (17.99)	1.21 (1.11, 1.33)^‡^	1.15 (1.05, 1.26)^†^
Inadequate	Inadequate	7,916 (14.03)	610 (16.58)	1.29 (1.17, 1.41)^‡^	1.14 (1.02, 1.28)^*^
*P* for interaction				0.0045	0.0001

^*^ *p* < 0.05; ^†^ *p* < 0.01; ^‡^ *p* < 0.001. PTB, preterm birth. ^a^ model was crude; ^b^ model was additionally adjusted with age, education, BMI, risk pregnancy, parity, calendar year of the first visit, alcohol or cigarette, and gestational age of the first visit.

Similarly, women had a higher risk of SGA if only the ANC5 (OR = 1.13, 95%CI = 1.06–1.22) or the APNCU (OR = 1.15, 95%CI = 1.00–1.31) were inadequate. No joint effect was detected (Table 5).

**Table 5:** Association between ANC5 and APNCU on SGA and LGA.

ANC5	APNCU	AGA	SGA			LGA		
		*N* (%)	*N* (%)	Model l ^a^	Model 2^b^	*N* (%)	Model 1^a^	Model 2^b^
				OR (95%CI)			OR (95%CI)	
Adequate	Adequate	31,102 (66.26)	4,430 (63.94)	Ref.	Ref.	4,045 (64.75)	Ref.	Ref.
Adequate	Inadequate	1716 (3.66)	268 (3.87)	1.10 (0.96, 1.25)	1.15 (1.00, 1.31)^*^	252 (4.03)	1.13 (0.99, 1.29)	1.11 (0.97, 1.28)
Inadequate	Adequate	7,532 (16.05)	1,185 (17.10)	1.10 (1.03, 1.18)^†^	1.13 (1.06, 1.22)^‡^	1,058 (16.94)	1.08 (1.00, 1.16)^*^	1.05 (0.97, 1.13)
Inadequate	Inadequate	6,589 (14.04)	1,045 (15.08)	1.11 (1.04, 1.20)^†^	1.16 (1.06, 1.26)^‡^	892 (14.28)	1.04 (0.96, 1.12)	1.00 (0.92, 1.10)
*P* for interaction				0.3032	0.1613		0.0623	0.0823

^*^p < 0.05; ^†^*p* < 0.01; ^‡^*p* < 0.001. AGA, appropriate for gestational age; SGA, small for gestational age; LGA, large for gestational age. ^a^ model was crude; ^b^ model was additionally adjusted age, education, BMI, risk pregnancy, parity, calendar year of the first visit, alcohol or cigarette, and gestational age of the first visit.

Supplementary Tables S4A,B shows the crossover analysis of prenatal care utilization measured in ANC5 and APNCU and birth weight and LBW/FM, respectively. To conclude, compared to women who met adequate criteria of both ANC5 and APNCU, women who completed adequate prenatal care only in APNCU were exposed to weight loss in newborns [*β* (se) = −42.29 (8.48)]; women were exposed to weight loss ANC5 was adequate [*β* (se) = −11.17 (4.51)]. An interaction between APNCU and ANC5 was detected in the effect of ANC5 and APNCU on birth weight. Women had a higher risk of LBW when mere inadequacy in APNCU (OR = 1.68, 95%CI: 1.19–2.39) or ANC5 (OR = 1.26, 95%CI: 1.07–1.50) was detected. An interaction was detected between ANC5 and APNCU (*p* < 0.05).

### 3.4 Sensitivity analysis

When changing BMI at the first visit to pre-pregnancy BMI, the association between prenatal care and adverse outcomes remained similar (Supplementary Tables S6A–S10B). In the stratification analysis (Supplementary Tables S11, S12), there was no significant difference in the association between prenatal care and adverse outcomes in different risk groups (*p* > 0.05).

## 4 Discussion

Our study indicated that inadequate ANC5 and APNCU would lead to birth weight loss and a higher risk of PTB, SGA, and LBW. The crossover analysis showed that women with inadequacies in either ANC5 or APNCU were more likely to deliver babies with small birth weight, LBW, and PTB. Additionally, ANC5 and APNCU had an interaction effect on PTB.

Our study revealed that inadequate prenatal care utilization was associated with a higher risk of PTB. This was in line with one previous study, which showed that prenatal care frequency was negatively related to PTB (23). As for APNCU, our result differed from previous studies (3), which found that women with inadequate prenatal care were exposed to a lower risk of PTB. One explanation could be that the distribution of needed prenatal care utilization in different trimesters was imbalanced, which means APNCU was likely to divide women with shorter gestational lengths into adequate plus category (7). The fact that approximately 80% of the women in our study population paid the first visit before or equal to the 12th gestational week means that the situation mentioned above may only appear in a small number of women. This could lead to a difference in association between APNCU and PTB.

Our study indicated that women paying less than five-time-specific visits had an elevated SGA risk. We also compared our results with previous studies in Brazil (15) and Mexico (24). Both studies suggested that women who paid more visits were less likely to have SGA babies. This differs from an earlier meta-analysis of randomized controlled trials (RCTs), which indicated that reduced ANC would not lead to a higher incidence of SGA (25). However, due to the drawback that the outcome analysis and allocation concealment could not be realized, the reliability of these analyses could be undermined. Besides, the conclusion of that RCT study was based on women with low-risk pregnancies, while there are high-risk women in the study population.

Similar to the result of ANC5, inadequate prenatal care in APNCU was associated with SGA. Our data also suggested that insufficient prenatal care influenced SGA births. Two studies indicated inadequate prenatal care usage was associated with SGA in America (21, 26). One study from America also showed no significant association between APNCU and SGA (18). The negative result could be because our study’s reference group was adequate plus and adequate groups combined. Moreover, those with inadequate prenatal care utilization and disproportionately multiparous mothers may also contribute to the differences observed in the study, even though prenatal care utilization was generally distributed evenly among women with different parities.

Different from preterm and SGA, ANC5 and APNCU showed no association with LBW and birth weight. However, previous studies have revealed that prenatal care frequency reduction (27) and prenatal care less than four times (28) were associated with birth weight loss. In addition, previous studies showed a negative association between prenatal care frequency and LBW (28, 29). This could be because the adequate definition of adequate prenatal care utilization in their study was relatively low- and high-level ANC was relatively low in the population, while the prenatal care level was relatively high in China. The protective effect of prenatal care utilization on LBW may disappear after the most basic prenatal care is completed.

The association between APNCU and LBW was similar to that between ANC5. There was no significant association between inadequate APNCU and LBW. This differs from previous studies (3, 30). Studies also showed that the risk of LBW was higher in the adequate plus and inadequate groups, with women in the adequate plus rank exposed to the highest level of LBW risk (2, 31). The difference in the result may arise from the confounders not being controlled.

A crossover analysis was applied to compare the effects of ANC5 and APNCU on birth weight, PTB, and LBW. What needs to be pointed out is that inadequate APNCU would only bring a higher risk of PTB and birth weight loss than inadequate ANC5. APNCU and ANC5 both showed independent risks for SGA and LBW. This means that although inadequate APNCU would bring more severe birth weight outcomes, ANC5 still influenced the outcome mentioned above in another pathway.

Previous studies have pointed out that there is a selection bias in prenatal care utilization. Specifically, the situations can be categorized into four types based on risk and utilization rates: high risk with high utilization, high risk with low utilization, low risk with high utilization, and low risk with low utilization. Therefore, we conducted a stratification analysis based on the risk of pregnancy. No stable interaction is detected between the risk pregnancy and the prenatal care utilization index. This is out of expectation since one previous study observed aggravation of the LBW outcome in women evaluated as having inadequate prenatal care but with a higher risk (3). One possible reason for the non-significant interaction is that high-risk pregnant women may have received more intensive medical interventions. These interventions could mitigate the differences in outcomes between high-risk and low-risk groups, leading to non-significant interaction terms. Effective management and care provided to high-risk pregnant women may blur the differences that might otherwise be observed. It could be due to the development of technology that fewer prenatal care visits may not necessarily result in worse outcomes. It means that we need to pay attention to both groups of women since they benefit similarly from prenatal care. It could also be that the low-risk standard was too strict. Therefore, it may underestimate the effect of prenatal care on birth outcomes.

Moreover, ANC5 was easier for health professionals to calculate and track and more straightforward for pregnant women to follow the medical advice from health professionals than APNCU. Few studies compared indices by crossover analysis. One similar study (18) described the association between prenatal care frequency and the original APNCU and SGA, respectively. It turned out that women with inadequate prenatal care, as measured by the original APNCU, were not exposed to a higher risk of SGA. This could be explained by the difference in the categorization of the index. Our study provided evidence that ANC5’s time-specific track of prenatal care utilization is necessary for women. It proves that ANC5 in China has its appropriateness. More evidence is needed to compare the difference between ANC5 and APNCU.

## 5 Strengths and limitations

One significant difficulty in identifying the effect of prenatal care on SGA is that prenatal care visits of women are also influenced by health status in each visit. Accordingly, our study’s strength lies in that women’s health was considered. We used doctors’ diagnoses and women’s symptoms to represent women’s health. Moreover, medical records were utilized to ensure the accuracy of the data collection.

There were also a few drawbacks to our study. First, only including women from Zhoushan could affect the population’s representativeness. Second, essential variables such as income and geographical information were not obtained while investigating the influencing factors, hence introducing confounding bias. Third, more outcomes were needed to compare the two indices.

## 6 Conclusion

Women with inadequate prenatal care in ANC5 or APNCU were more likely to suffer from adverse birth outcomes, including PTB, birth weight loss, SGA, and LBW. It indicates that adequate prenatal care is necessary for pregnant women. However, ANC5 and APNCU had an interaction with PTB, and only inadequate prenatal care use by APNCU had the highest risk of PTB, which indicates that APNCU would be a better tool for evaluating prenatal care use.

## Data availability statement

The data presented in this study are available on request from the corresponding author. The data are not publicly available because they contain information that could compromise the privacy of research participants.

## Ethics statement

The studies involving humans were approved by the Zhejiang University School of Medicine. The studies were conducted in accordance with the local legislation and institutional requirements. The participants provided their written informed consent to participate in this study.

## Author contributions

HZ: Writing – original draft. PC: Formal analysis, Writing – original draft. HC: Writing – review & editing. XA: Methodology, Writing – original draft. YQ: Data curation, Software, Writing – original draft. YH: Investigation, Writing – review & editing. LZ: Resources, Writing – review & editing. DA: Supervision, Writing – review & editing. YZ: Validation, Writing – review & editing. HL: Software, Writing – review & editing. ZC: Investigation, Writing – review & editing. YuY: Conceptualization, Writing – review & editing. YiY: Writing – review & editing.
