# Supplementary material for: The association of Chinese and American antenatal care utilization indices with birth outcomes
Source: Front Public Health. 2024 Aug 7;12:1420943. doi: 10.3389/fpubh.2024.1420943 (PMC11335484; doi:10.3389/fpubh.2024.1420943)
Supplement: Supplementary file 1 [file Data_Sheet_1.docx]

**Supplementary**

Figure S1. Data processing flow


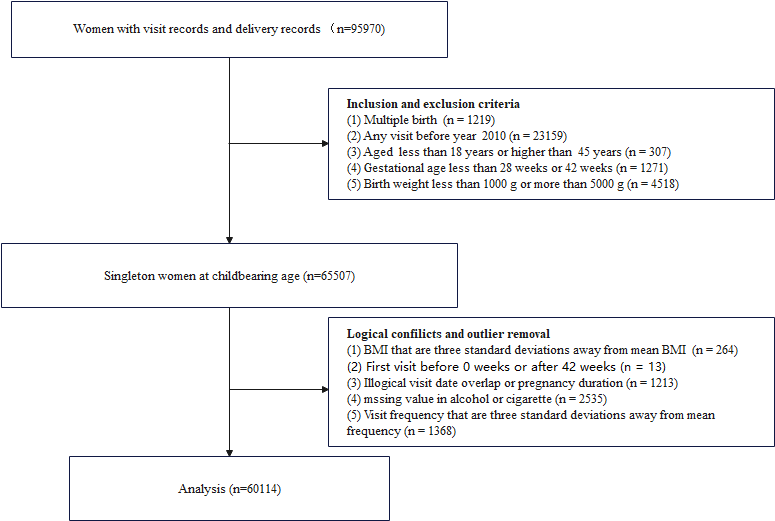


Figure S2. Restricted cublic spline of prenatal care visit frequency


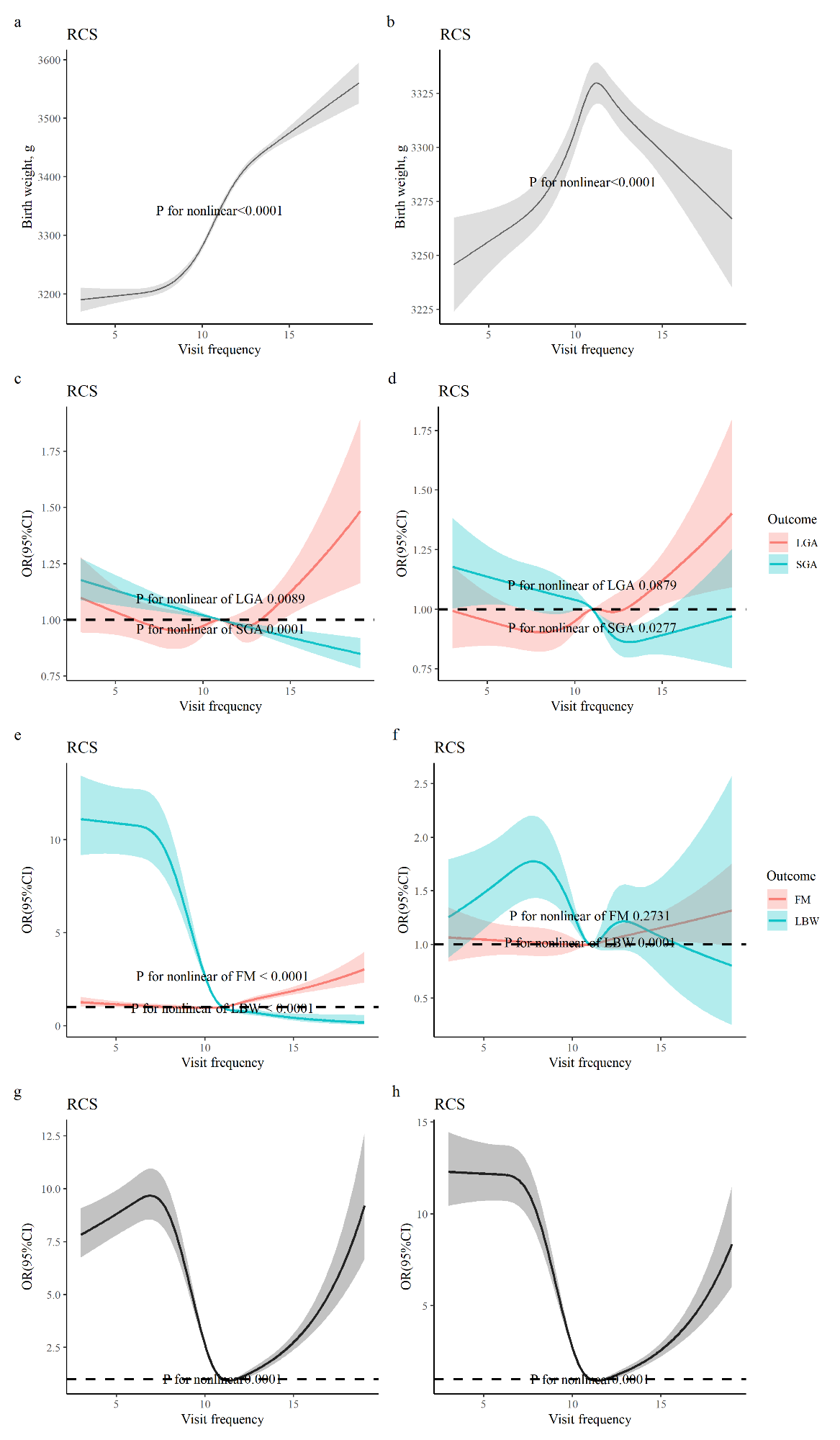


RCS of visit frequency and adverse birth outcome a) RCS of visit frequency and birth weight with no additional variables adjusted. b) RCS of visit frequency and birth weight with age, education, BMI, risk diagnosis, parity, calendar year of the first visit, alcohol or cigarette, gestational age of the first visit, gestational age of delivery additionally adjusted. c) RCS of visit frequency and SGA/LGA. d) RCS of visit frequency with age, education, BMI, risk diagnosis, parity, calendar year of the first visit, alcohol or cigarette, gestational age of the first visit, gestational age of delivery additionally adjusted. e) RCS of visit frequency and LBW/FM. f) RCS of visit frequency and LBW/FM with age, education, BMI, risk diagnosis, parity, calendar year of the first visit, alcohol or cigarette, gestational age of the first visit and gestational age of delivery additionally adjusted. g) RCS of visit frequency and preterm. h) RCS of visit frequency and preterm with age, education, BMI, risk diagnosis, parity, calendar year of the first visit, gestational age of the first visit and alcohol or cigarette additionally adjusted.

| **Table S1. High risk diagnosis criteria** |
| --- |
| **Symptoms or medical history** |
| History of cesarean delivery (1 time) |
| Uterine fibroids removal surgery interval less than 1 year |
| Excessive amniotic fluid |
| Premature rupture of membranes before 34th week |
| Premature rupture of membranes between 34th and 37th week |
| Premature birth before 34th week |
| Premature birth after 34th week |
| Immune system disease active period |
| Immune system disease quiescence |
| Acute appendicitis |
| Epilepsy requiring pharmacological control |
| Episodes of epilepsy |
| Stable thyroid disease |
| History of heart failure |
| Unstable phase of mental illness |
| Stable mental illness |
| Malformation of the birth canal |
| cesarean section interval less than 1 year |
| History of more than 3 times of induced abortions |
| BMI >24 (first trimester) |
| Weight <40kg (first trimester) |
| Aged more than 35 years by due date |
| Post-organ transplantation |
| Deafness |
| Gestational trophoblastic disease |
| Gigantic baby |
| Fetal growth restriction |
| Fetal malformation |
| Intrauterine fetal death |
| Pregnancy at term |
| Fetal distress (NST ≤ 7) |
| Hyperemesis gravidarum |
| Ectopic pregnancy |
| Possible Rh blood group incompatibility |
| History of multiple blood transfusions prior to pregnancy |
| Multiple pregnancies (three or more) |
| twin pregnancies |
| abnormal fetal position after or equal to 36 weeks |
| Severe (bile acids ≥40umol/l) |
| Mild (bile acids ≥11umol/l, <40umol/l) |
| Primary hypertension complicated by pre-eclampsia |
| HELLP syndrome |
| Eclampsia |
| Severe pre-eclampsia |
| Primary hypertension (BP ≥ 140/90 mmHg) |
| Pre-eclampsia (mild) |
| Hypertension during pregnancy |
| Central placenta praevia, placenta abruption |
| Placenta praevia (marginal, partial) |
| Placenta praevia (low lying) |
| Acute amniotic fluid overload |
| Umbilical cord prolapse |
| Umbilical cord wrapping around the neck (≥2 turns) |
| Life-threatening malignancy |
| Unexplained high fever, etc. |
| Severe medical illness: severe infection |
| Serious medical illness: stroke |
| Serious medical illness: coma |
| Acute and chronic urinary tract infection |
| Appendicitis (stable stage) |
| Positive HIV Antibody Confirmation |
| Positive HIV antibody test |
| Gonorrhea |
| Syphilis untreated |
| Syphilis currently on standard treatment |
| Moderate to severe pulmonary insufficiency |
| With mild pulmonary insufficiency |
| Normal lung function |
| hyperthyroidism in critical condition |
| Unstable thyroid disease |
| Severe complications of diabetes mellitus |
| Grade A2 diabetes mellitus |
| Grade A1 diabetes mellitus |
| Very severe anemia (Hb≤30g/l) |
| Thrombocytopenia (PLT <50×109/L) |
| Evans' (Evans) syndrome (autoimmune anemia combined with thrombocytopenia) |
| Severe hematologic disorders: severe reoccurrence |
| Moderate anemia (Hb61-80g/l) |
| Mild anemia (Hb 81 to 100g/l) |
| Thrombocytopenia (PLT 100 to 70×109/l) |
| Cornual tuberculosis |
| Open pulmonary tuberculosis |
| Tuberculosis (active type) |
| Pulmonary tuberculosis (stable type) |
| Chronic kidney disease with severe hypertension (BP ≥ 160/110 mmHg) or proteinuria |
| Renal disease (with mild renal impairment) |
| Renal disease (normal renal function) |
| Severe hepatitis |
| Cirrhosis of the liver |
| Liver damage (ALT ≥ 100 U/L) |
| Viral hepatitis (ALT≥100U/L) |
| Viral hepatitis |
| Atrial fibrillation |
| Ventricular fibrillation |
| History of cardiogenic shock |
| Third-degree atrioventricular block |
| Severe arrhythmia |
| right-to-left shunt precordial |
| Pulmonary hypertension |
| Atrioventricular block |
| Frequent premature beats |
| History of cardiac surgery |
| Myocarditis sequelae |
| Heart disease:Class I-II cardiac function |
| Maternal limitations in health care and access to care due to family or social reasons |
| Viral infection in early pregnancy |
| Exposure to harmful physical and chemical factors in early pregnancy |
| Spinal deformity |
| Thoracic deformity with moderate to severe pulmonary insufficiency |
| Thoracic deformity with mild pulmonary insufficiency |
| Thoracic deformity without pulmonary insufficiency |
| Severe hereditary disease |
| Pregnant women and first-degree relatives with a history of genetic disorders |
| Assisted reproduction |
| High risk |
| Moderate risk |
| Mild risk |
| History of uterine rupture |
| Height ≤ 145cm |
| Low amniotic fluid |
| Loose endocervical opening |
| Narrow pelvis |
| History of more than 2 times of cesarean deliveries |
| History of preterm birth |
| stillbirth |
| Stillbirth |
| BMI <18 |
| Weight ≥80Kg (first trimester) |
| Age <18 years according to expected date of delivery |
| History of uterine fibroid excavation interval ≥2 years |
| Severe anemia (Hb 31-60g/l) |
| Thrombocytopenia (PLT 70-50×109/L) |
| Renal insufficiency |
| Acute fatty liver |
| Liver damage (ALT <100U/L) |
| Rheumatic fever active stage |
| Severe cardiac lesions:Cardiac function class III-IV |
| Hypertension before 19th week |
| Gstational diabetes mellitus |
| Hypertension after 20th week |
| Abbreviation: BMI, Body Mass Index; BP, blood pressure; NST, none-stress test; BP, blood pressure; Hb, Hemoglobin; PLT, blood platelet; ALT, alanine transaminase. |

| **Table S2. Distribution of socio-economic characteristics** | | | | | |
| --- | --- | --- | --- | --- | --- |
| **Variable** | **ANC5** | |  | **APNCU** | |
|  | **Adequate** | **Inadequate** |  | **Adequate** | **Inadequate** |
|  | **(n = 41813)** | **(n = 18301)** |  | **(n = 49352)** | **(n = 10762)** |
|  | **Mean ± SD** |  |  |  |  |
| **Age** | 28.47 ± 4.00 | 28.22 ± 4.69 ^‡^ |  | 28.51 ± 4.09 | 27.87 ± 4.75 ^‡^ |
| **Gestational age of delivery** | 39.07 ± 1.48 | 39.15 ± 1.52 ^‡^ |  | 39.02 ± 1.49 | 39.43 ± 1.47 ^‡^ |
| **Gestational age of the first visit** | 11.18 ± 1.22 | 14.39 ± 5.08 ^‡^ |  | 11.55 ± 1.70 | 14.96 ± 6.25 ^‡^ |
|  | **N (%)** |  |  |  |  |
| **Education** |  | ^‡^ |  |  | ^‡^ |
| Middle school or less | 8610 (20.6) | 6887 (37.6) |  | 11076 (22.4) | 4421 (41.1) |
| High school | 8002 (19.1) | 3455 (18.9) |  | 9547 (19.3) | 1910 (17.7) |
| College or more | 25201 (60.3) | 7959 (43.5) |  | 28729 (58.2) | 4431 (41.2) |
| **BMI at the first visit** |  | ^‡^ |  |  | ^‡^ |
| Underweight | 6686 (16.0) | 2148 (11.7) |  | 7578 (15.4) | 1256 (11.7) |
| Normal | 28491 (68.1) | 12035 (65.8) |  | 33565 (68.0) | 6961 (64.7) |
| Overweight | 5312 (12.7) | 3183 (17.4) |  | 6522 (13.2) | 1973 (18.3) |
| Obesity | 1324 (3.2) | 935 (5.1) |  | 1687 (3.4) | 572 (5.3) |
| **Risk pregnancy** |  | ^‡^ |  |  | ^‡^ |
| Low risk | 16023 (38.3) | 7954 (43.5) |  | 18718 (37.9) | 5259 (48.9) |
| High risk | 25790 (61.7) | 10347 (56.5) |  | 30634 (62.1) | 5503 (51.1) |
| **Parity** |  | ^‡^ |  |  | ^‡^ |
| Primipara | 30601 (73.2) | 10714 (58.5) |  | 35282 (71.5) | 6033 (56.1) |
| Multipara | 11212 (26.8) | 7587 (41.5) |  | 14070 (28.5) | 4729 (43.9) |
| **Calendar year of the first visit** |  | ^‡^ |  |  | ^‡^ |
| 2010 ~ | 9163 (21.9) | 3370 (18.4) |  | 10566 (21.4) | 1967 (18.3) |
| 2013 ~ | 11258 (26.9) | 5723 (31.3) |  | 13303 (27.0) | 3678 (34.2) |
| 2016 ~ | 10489 (25.1) | 4964 (27.1) |  | 12389 (25.1) | 3064 (28.5) |
| 2019 ~ | 10903 (26.1) | 4244 (23.2) |  | 13094 (26.5) | 2053 (19.1) |
| **Alcohol or cigarette** |  | ^‡^ |  |  |  |
| No | 41299 (98.8) | 18004 (98.4) |  | 48704 (98.7) | 10599 (98.5) |
| Yes | 514 (1.2) | 297 (1.6) |  | 648 (1.3) | 163 (1.5) |
| **original APNCU** |  | ^‡^ |  |  |  |
| Adequate plus | 8726 (20.9) | 1354 (7.4) |  | - | - |
| Adequate | 30851 (73.8) | 8421 (46.0) |  | - | - |
| Intermediate | 2204 (5.3) | 5007 (27.4) |  | - | - |
| Inadequate | 32 (0.1) | 3519 (19.2) |  | - | - |
| **ANC5** |  |  |  |  | ^‡^ |
| Adequate | - | - |  | 39577 (80.2) | 2236 (20.8) |
| Inadequate | - | - |  | 9775 (19.8) | 8526 (79.2) |
| **APNCU** |  | ^‡^ |  |  |  |
| Adequate | 39577 (94.7) | 9775 (53.4) |  | - | - |
| Inadequate | 2236 (5.3) | 8526 (46.6) |  | - | - |
| ^*^ P < 0.05; ^†^ P < 0.01; ^‡^ P < 0.001; Comparison was made within APNCU and ANC5 respectively | | | | | |

| **Table S3a. Association between prenatal care and birthweight** | | | | |
| --- | --- | --- | --- | --- |
| **Variable** | **N (%)** |  | **Model 1 ^a^** | **Model 2 ^b^** |
|  |  |  | **β (se)** | |
| **Prenatal care visit frequency** |  |  |  |  |
| 0 ~ | 22232 (36.98) |  | Ref. | Ref. |
| 11 ~ | 10871 (18.08) |  | 95.89 (5.19) ^‡^ | 34.36 (4.70) ^‡^ |
| 12 ~ | 19855 (33.03) |  | 164.07 (4.33) ^‡^ | 26.86 (4.22) ^‡^ |
| 14 ~ | 7156 (11.90) |  | 228.07 (6.02) ^‡^ | 7.93 (5.88) |
| **ANC5** |  |  |  |  |
| Adequate | 41813 (69.56) |  | Ref. | Ref. |
| Inadequate | 18301 (30.44) |  | -0.12 (4.00) | -14.21 (3.87) ^‡^ |
| **APNCU** |  |  |  |  |
| Adequate | 49352 (82.10) |  | Ref. | Ref. |
| Inadequate | 10762 (17.90) |  | 37.69 (4.79) ^‡^ | -26.68 (4.57) ^‡^ |
| ^*^ P < 0.05; ^†^ P < 0.01; ^‡^ P < 0.001; ^a^ Model was crude ^b^ Model was adjusted with age, education, BMI, high risk pregnancy, parity, calendar year of the first visit, alcohol or cigarette, and gestational age of the first visit. | | | | |

| **Table S3b. Association between prenatal care and LBW and macrosomia** | | | | | | | | | |
| --- | --- | --- | --- | --- | --- | --- | --- | --- | --- |
| **Variable** | **Normal** |  | **LBW** | | |  | **Macrosomia** | | |
|  | **N (%)** |  | **N (%)** | **Model 1 ^a^** | **Model 2 ^b^** |  | **N (%)** | **Model 1 ^a^** | **Model 2 ^b^** |
|  |  |  |  | **OR (95%CI)** | |  |  | **OR (95%CI)** | |
| **Prenatal care visit frequency** |  |  |  |  |  |  |  |  |  |
| 0 ~ | 19827 (36.13) |  | 1342 (80.60) | Ref. | Ref. |  | 1063 (29.73) | Ref. | Ref. |
| 11 ~ | 10154 (18.50) |  | 145 (8.71) | 0.21 (0.18, 0.25) ^‡^ | 0.76 (0.63, 0.93) ^†^ |  | 572 (16.00) | 1.05 (0.95, 1.17) | 1.04 (0.93, 1.16) |
| 12 ~ | 18418 (33.56) |  | 145 (8.71) | 0.12 (0.10, 0.14) ^‡^ | 0.79 (0.64, 0.96) ^*^ |  | 1292 (36.13) | 1.31 (1.20, 1.42) ^‡^ | 1.06 (0.96, 1.16) |
| 14 ~ | 6474 (11.80) |  | 33 (1.98) | 0.08 (0.05, 0.11) ^‡^ | 0.76 (0.51, 1.12) |  | 649 (18.15) | 1.87 (1.69, 2.07) ^‡^ | 1.18 (1.05, 1.32) ^†^ |
| **ANC5** |  |  |  |  |  |  |  |  |  |
| Adequate | 38215 (69.64) |  | 1142 (68.59) | Ref. | Ref. |  | 2456 (68.68) | Ref. | Ref. |
| Inadequate | 16658 (30.36) |  | 523 (31.41) | 1.05 (0.95, 1.17) | 1.14 (0.98, 1.34) |  | 1120 (31.32) | 1.05 (0.97, 1.13) | 1.00 (0.92, 1.09) |
| **APNCU** |  |  |  |  |  |  |  |  |  |
| Adequate | 45069 (82.13) |  | 1423 (85.47) | Ref. | Ref. |  | 2860 (79.98) | Ref. | Ref. |
| Inadequate | 9804 (17.87) |  | 242 (14.53) | 0.78 (0.68, 0.90) ^‡^ | 1.04 (0.85, 1.27) |  | 716 (20.02) | 1.15 (1.06, 1.25) ^†^ | 0.96 (0.88, 1.06) |
| ^*^ P < 0.05; ^†^ P < 0.01; ^‡^ P < 0.001; ^a^ Model was crude ^b^ Model was additionally adjusted for age, education, BMI, high risk diagnosis, parity, calendar year of the first visit, alcohol or cigarette, gestational age of the first visit, and gestational age of delivery. | | | | | | | | | |

| **Table S4a. Crossover analysis of ANC5 and APNCU on birth weight** | | | | | |
| --- | --- | --- | --- | --- | --- |
| **ANC5** | **APNCU** | **N (%)** |  | **Model 1 ^a^** | **Model 2 ^b^** |
|  |  |  |  | **β (se)** | |
| Adequate | Adequate | 39577 (65.84) |  | Ref. | Ref. |
| Adequate | Inadequate | 2236 (3.72) |  | 70.44 (9.79) ‡ | -42.29 (8.48) ‡ |
| Inadequate | Adequate | 9775 (16.26) |  | -15.24 (5.09) † | -11.17 (4.51) * |
| Inadequate | Inadequate | 8526 (14.18) |  | 25.30 (5.38) ‡ | -25.65 (5.41) ‡ |
| P for interaction |  |  |  | 0.0116 | 0.0071 |
| ^*^ P < 0.05; ^†^ P < 0.01; ^‡^ P < 0.001; ^a^ Model was crude ^b^ Model was adjusted with age, education, BMI, high risk pregnancy, parity, calendar year of the first visit, alcohol or cigarette, gestational age of the first visit, and gestational age of delivery. | | | | | |

| **Table S4b. Crossover analysis of ANC5 and APNCU on LBW and Macrosomia** | | | | | | | | | | |
| --- | --- | --- | --- | --- | --- | --- | --- | --- | --- | --- |
| **ANC5** | **APNCU** | **Normal** |  | **LBW** | | |  | **Macrosomia** | | |
|  |  | **N (%)** |  | **N (%)** | **Model 1 ^a^** | **Model 2 ^b^** |  | **N (%)** | **Model 1 ^a^** | **Model 2 ^b^** |
|  |  |  |  |  | **OR (95%CI)** | |  |  | **OR (95%CI)** | |
| Adequate | Adequate | 36214 (66.00) |  | 1091 (65.53) | Ref. | Ref. |  | 2272 (63.53) | Ref. | Ref. |
| Adequate | Inadequate | 2001 (3.65) |  | 51 (3.06) | 0.85 (0.64, 1.12) | 1.68 (1.19, 2.39) ^†^ |  | 184 (5.15) | 1.47 (1.25, 1.71) ^‡^ | 1.07 (0.91, 1.26) |
| Inadequate | Adequate | 8855 (16.14) |  | 332 (19.94) | 1.24 (1.10, 1.41) ^‡^ | 1.26 (1.07, 1.50) ^†^ |  | 588 (16.44) | 1.06 (0.96, 1.16) | 1.05 (0.95, 1.16) |
| Inadequate | Inadequate | 7803 (14.22) |  | 191 (11.47) | 0.81 (0.70, 0.95) ^†^ | 0.96 (0.75, 1.23) |  | 532 (14.88) | 1.09 (0.99, 1.20) | 0.94 (0.84, 1.06) |
| P for interaction |  |  |  |  | 0.1314 | 0.0003 |  |  | 0.0004 | 0.0791 |
| ^*^ P < 0.05; ^†^ P < 0.01; ^‡^ P < 0.001; ^a^  Model was crude; ^b^ Model was adjusted with age, education, BMI, high risk pregnancy, parity, calendar year of the first visit, alcohol or cigarette, gestational age of the first visit, and gestational age of delivery. | | | | | | | | | | |

| **Table S5. Distribution of socio-economic characteristics** | |
| --- | --- |
| **Variable** | **Overall (n = 6601)** |
|  | Mean ± SD |
| **Age** | 28.96 ± 3.79 |
| **Gestational age of delivery** | 39.13 ± 1.53 |
| **Gestational age of the first visit** | 11.52 ± 1.13 |
|  | N (%) |
| **Education** |  |
| Middle school or less | 515 (7.8) |
| High school | 1104 (16.7) |
| College or more | 4982 (75.5) |
| **BMI at the first visit** |  |
| Underweight | 1031 (15.6) |
| Normal | 4601 (69.7) |
| Overweight | 804 (12.2) |
| Obesity | 165 (2.5) |
| **Pre-pregnancy BMI** |  |
| Underweight | 1321 (20.0) |
| Normal | 4472 (67.8) |
| Overweight | 667 (10.1) |
| Obesity | 140 (2.1) |
| **Risk pregnancy** |  |
| Low risk | 1842 (27.9) |
| High risk | 4759 (72.1) |
| **Parity** |  |
| Primipara | 5169 (78.3) |
| Multipara | 1432 (21.7) |
| **Calendar year of the first visit** |  |
| 2010 ~ | 1439 (21.8) |
| 2013 ~ | 1749 (26.5) |
| 2016 ~ | 1855 (28.1) |
| 2019 ~ | 1558 (23.6) |
| **Alcohol or cigarette** |  |
| No | 6532 (99.0) |
| Yes | 69 (1.0) |
| **original APNCU** |  |
| Adequate plus | 1074 (16.3) |
| Adequate | 4947 (74.9) |
| Intermediate | 528 (8.0) |
| Inadequate | 52 (0.8) |
| **ANC5** |  |
| Adequate | 5382 (81.5) |
| Inadequate | 1219 (18.5) |
| **APNCU** |  |
| Adequate | 6021 (91.2) |
| Inadequate | 580 (8.8) |

| **Table S6a. Association between prenatal care and PTB** | | | | | |
| --- | --- | --- | --- | --- | --- |
| **Variable** | **PTB** | |  | **Model 1 ^a^** | **Model 2 ^b^** |
|  | **No** | **Yes** |  | **OR (95%CI)** | |
| **Prenatal care visit frequency** |  |  |  |  |  |
| Q1(1 ~) | 1550 (25.18) | 278 (62.33) |  | Ref. | Ref. |
| Q2(11 ~) | 2777 (45.12) | 74 (16.59) |  | 0.13 (0.10, 0.18) ^‡^ | 0.14 (0.10, 0.18) ^‡^ |
| Q3(13 ~) | 1036 (16.83) | 42 (9.42) |  | 0.21 (0.15, 0.29) ^‡^ | 0.21 (0.15, 0.29) ^‡^ |
| Q4(14 ~) | 792 (12.87) | 52 (11.66) |  | 0.34 (0.25, 0.47) ^‡^ | 0.35 (0.26, 0.48) ^‡^ |
| **ANC5** |  |  |  |  |  |
| Adequate | 5036 (81.82) | 346 (77.58) |  | Ref. | Ref. |
| Inadequate | 1119 (18.18) | 100 (22.42) |  | 0.94 (0.72, 1.22) | 0.94 (0.72, 1.23) |
| **APNCU** |  |  |  |  |  |
| Adequate | 5641 (91.65) | 380 (85.2) |  | Ref. | Ref. |
| Inadequate | 514 (8.35) | 66 (14.8) |  | 1.96 (1.46, 2.63) ^‡^ | 1.96 (1.46, 2.63) ^‡^ |
| ^*^ P < 0.05; ^†^ P < 0.01; ^‡^ P < 0.001; ^a^ Model was adjusted with age, education, pre-pregnancy BMI, risk pregnancy, parity, calendar year of the first visit, alcohol or cigarette, and gestational age of the first visit; ^b^ Model was adjusted with age, education, BMI, risk pregnancy, parity, calendar year of the first visit, alcohol or cigarette, and gestational age of the first visit. | | | | | |

| **Table S6b. Association between prenatal care and SGA and LGA** | | | | | | | | | |
| --- | --- | --- | --- | --- | --- | --- | --- | --- | --- |
| **Variable** | **APA** |  | **SGA** | | |  | **LGA** | | |
|  | **N (%)** |  | **N (%)** | **Model 1 ^a^** | **Model 2 ^b^** |  | **N (%)** | **Model 1 ^a^** | **Model 2 ^b^** |
|  |  |  |  | **OR (95%CI)** | |  |  | **OR (95%CI)** | |
| **Prenatal care visit frequency** |  |  |  |  |  |  |  |  |  |
| Q1(1 ~) | 1405 (27.35) |  | 258 (30.64) | Ref. | Ref. |  | 165 (26.57) | Ref. | Ref. |
| Q2(11 ~) | 2234 (43.48) |  | 362 (42.99) | 0.83 (0.70, 0.99) ^*^ | 0.82 (0.69, 0.98) ^*^ |  | 255 (41.06) | 1.02 (0.83, 1.26) | 1.03 (0.83, 1.27) |
| Q3(13 ~) | 856 (16.66) |  | 120 (14.25) | 0.72 (0.57, 0.91) ^†^ | 0.72 (0.57, 0.92) ^†^ |  | 102 (16.43) | 1.04 (0.79, 1.36) | 1.04 (0.79, 1.36) |
| Q4(14 ~) | 643 (12.51) |  | 102 (12.11) | 0.82 (0.64, 1.06) | 0.82 (0.64, 1.06) |  | 99 (15.94) | 1.33 (1.01, 1.75) ^*^ | 1.33 (1.01, 1.75) ^*^ |
| **ANC5** |  |  |  |  |  |  |  |  |  |
| Adequate | 4211 (81.96) |  | 670 (79.57) | Ref. | Ref. |  | 501 (80.68) | Ref. | Ref. |
| Inadequate | 927 (18.04) |  | 172 (20.43) | 1.14 (0.94, 1.39) | 1.14 (0.94, 1.39) |  | 120 (19.32) | 1.12 (0.90, 1.40) | 1.12 (0.90, 1.40) |
| **APNCU** |  |  |  |  |  |  |  |  |  |
| Adequate | 4691 (91.30) |  | 758 (90.02) | Ref. | Ref. |  | 572 (92.11) | Ref. | Ref. |
| Inadequate | 447 (8.70) |  | 84 (9.98) | 1.25 (0.97, 1.62) | 1.26 (0.98, 1.63) |  | 49 (7.89) | 0.85 (0.62, 1.17) | 0.84 (0.61, 1.16) |
| ^*^ P < 0.05; ^†^ P < 0.01; ^‡^ P < 0.001; ^a^ Model was adjusted for age, education, pre-pregnancy BMI, risk pregnancy, parity, calendar year of the first visit, alcohol or cigarette and gestational age of the first visit^b^ Model was adjusted for age, education, BMI, risk pregnancy, parity, calendar year of the first visit, alcohol or cigarette and gestational age of the first visit. | | | | | | | | | |

| **Table S7a. Crossover analysis of APNCU on PTB** | | | | | | |
| --- | --- | --- | --- | --- | --- | --- |
| **ANC5** | **APNCU** | **PTB** | |  | **Model 1^b^** | **Model 2** |
|  |  | **No** | **Yes** |  | **OR (95%CI)** | |
| Adequate | Adequate | 4846 (78.73) | 319 (71.52) |  | Ref. | Ref. |
| Adequate | Inadequate | 190 (3.09) | 27 (6.05) |  | 2.51 (1.60, 3.81) ^‡^ | 2.51 (1.60, 3.80) ^‡^ |
| Inadequate | Adequate | 795 (12.92) | 61 (13.68) |  | 0.80 (0.58, 1.09) | 0.81 (0.58, 1.10) |
| Inadequate | Inadequate | 324 (5.26) | 39 (8.74) |  | 1.56 (1.05, 2.27) ^*^ | 1.56 (1.05, 2.26) ^*^ |
| ***P***_interaction_ |  |  |  |  | 0.4187 | 0.4050 |
| ^*^ P < 0.05; ^†^ P < 0.01; ^‡^ P < 0.001; ^a^ Model was adjusted with age, education, pre-pregnancy, risk pregnancy, parity, calendar year of the first visit, alcohol or cigarette, and gestational age of the first visit; ^b^ Model was adjusted with age, education, BMI, risk pregnancy, parity, calendar year of the first visit, alcohol or cigarette, and gestational age of the first visit; | | | | | | |

| **Table S7b. Association between ANC5 and APNCU on SGA and LGA** | | | | | | | | | | |
| --- | --- | --- | --- | --- | --- | --- | --- | --- | --- | --- |
| **ANC5** | **APNCU** | **AGA** |  | **SGA** | | |  | **LGA** | | |
|  |  | **N (%)** |  | **N (%)** | **Model 1 ^a^** | **Model 2 ^b^** |  | **N (%)** | **Model 1 ^a^** | **Model 2 ^b^** |
|  |  |  |  |  | **OR (95%CI)** | |  |  | **OR (95%CI)** | |
| Adequate | Adequate | 4048 (78.79) |  | 638 (75.77) | Ref. | Ref. |  | 479 (77.13) | Ref. | Ref. |
| Adequate | Inadequate | 163 (3.17) |  | 32 (3.80) | 1.38 (0.93, 2.05) | 1.37 (0.92, 2.04) |  | 22 (3.54) | 1.08 (0.68, 1.72) | 1.09 (0.68, 1.73) |
| Inadequate | Adequate | 643 (12.51) |  | 120 (14.25) | 1.14 (0.91, 1.42) | 1.13 (0.90, 1.41) |  | 93 (14.98) | 1.30 (1.01, 1.66)* | 1.31 (1.02, 1.67)* |
| Inadequate | Inadequate | 284 (5.53) |  | 52 (6.18) | 1.23 (0.89, 1.69) | 1.25 (0.91, 1.71) |  | 27 (4.35) | 0.78 (0.52, 1.18) | 0.76 (0.50, 1.15) |
| ***P***_interaction_ |  |  |  |  |  | 0.4232 |  |  |  | 0.0603 |
| ^*^ P < 0.05; ^†^ P < 0.01; ^‡^ P < 0.001; ^a^  Model was adjusted with age, education, pre-pregnancy BMI, risk pregnancy, parity, calendar year of the first visit, alcohol or cigarette, and gestational age of the first visit; ^b^ Model was adjusted with age, education, BMI, risk pregnancy, parity, calendar year of the first visit, alcohol or cigarette, and gestational age of the first visit | | | | | | | | | | |

| **Table S8. Distribution of socio-economic characteristics** | | | | | |
| --- | --- | --- | --- | --- | --- |
| **Variable** | **ANC5** | |  | **APNCU** | |
|  | **Adequate** | **Inadequate** |  | **Adequate** | **Inadequate** |
|  | **(n = 5382)** | **(n = 1219)** |  | **(n = 6021)** | **(n = 580)** |
|  | **Mean ± SD** |  |  |  |  |
| **Age** | 28.92 ± 3.78 | 29.15 ± 3.86 |  | 29.00 ± 3.80 | 28.59 ± 3.73 ^*^ |
| **Gestational age of delivery** | 39.10 ± 1.53 | 39.22 ± 1.53 ^*^ |  | 39.07 ± 1.52 | 39.68 ± 1.50 ^‡^ |
| **Gestational age of the first visit** | 11.39 ± 0.88 | 12.13 ± 1.76 ^‡^ |  | 11.50 ± 1.07 | 11.73 ± 1.67 ^‡^ |
|  | **N (%)** |  |  |  |  |
| **Education** |  |  |  |  | ^*^ |
| Middle school or less | 412 (7.7) | 103 (8.4) |  | 453 (7.5) | 62 (10.7) |
| High school | 881 (16.4) | 223 (18.3) |  | 999 (16.6) | 105 (18.1) |
| College or more | 4089 (76.0) | 893 (73.3) |  | 4569 (75.9) | 413 (71.2) |
| **BMI at the first visit** |  |  |  |  |  |
| Underweight | 865 (16.1) | 166 (13.6) |  | 931 (15.5) | 100 (17.2) |
| Normal | 3748 (69.6) | 853 (70.0) |  | 4202 (69.8) | 399 (68.8) |
| Overweight | 640 (11.9) | 164 (13.5) |  | 733 (12.2) | 71 (12.2) |
| Obesity | 129 (2.4) | 36 (3.0) |  | 155 (2.6) | 10 (1.7) |
| **Pre-pregnancy BMI** |  |  |  |  |  |
| Underweight | 1110 (20.6) | 211 (17.3) |  | 1187 (19.7) | 134 (23.1) |
| Normal | 3627 (67.4) | 845 (69.3) |  | 4092 (68.0) | 380 (65.5) |
| Overweight | 533 (9.9) | 134 (11.0) |  | 611 (10.1) | 56 (9.7) |
| Obesity | 111 (2.1) | 29 (2.4) |  | 130 (2.2) | 10 (1.7) |
| **Risk pregnancy** |  |  |  |  | ^‡^ |
| Low risk | 1502 (27.9) | 340 (27.9) |  | 1605 (26.7) | 237 (40.9) |
| High risk | 3880 (72.1) | 879 (72.1) |  | 4416 (73.3) | 343 (59.1) |
| **Parity** |  | ^*^ |  |  |  |
| Primipara | 4243 (78.8) | 926 (76.0) |  | 4727 (78.5) | 442 (76.2) |
| Multipara | 1139 (21.2) | 293 (24.0) |  | 1294 (21.5) | 138 (23.8) |
| **Calendar year of the first visit** |  | ^‡^ |  |  | ^‡^ |
| 2010 ~ | 1217 (22.6) | 222 (18.2) |  | 1289 (21.4) | 150 (25.9) |
| 2013 ~ | 1346 (25.0) | 403 (33.1) |  | 1471 (24.4) | 278 (47.9) |
| 2016 ~ | 1559 (29.0) | 296 (24.3) |  | 1764 (29.3) | 91 (15.7) |
| 2019 ~ | 1260 (23.4) | 298 (24.4) |  | 1497 (24.9) | 61 (10.5) |
| **Alcohol or cigarette** |  |  |  |  |  |
| No | 5331 (99.1) | 1201 (98.5) |  | 5960 (99.0) | 572 (98.6) |
| Yes | 51 (0.9) | 18 (1.5) |  | 61 (1.0) | 8 (1.4) |
| **original APNCU** |  | ^‡^ |  |  |  |
| Adequate plus | 994 (18.5) | 80 (6.6) |  | - | - |
| Adequate | 4171 (77.5) | 776 (63.7) |  | - | - |
| Intermediate | 215 (4.0) | 313 (25.7) |  | - | - |
| Inadequate | 2 (0.0) | 50 (4.1) |  | - | - |
| **ANC5** |  |  |  |  | ^‡^ |
| Adequate | - | - |  | 5165 (85.8) | 217 (37.4) |
| Inadequate | - | - |  | 856 (14.2) | 363 (62.6) |
| **APNCU** |  | ^‡^ |  |  |  |
| Adequate | 5165 (96.0) | 856 (70.2) |  | - | - |
| Inadequate | 217 (4.0) | 363 (29.8) |  | - | - |
| ^*^ P < 0.05; ^†^ P < 0.01; ^‡^ P < 0.001; Comparison was made within APNCU and ANC5 respectively | | | | | |

| **Table S9a. Association between prenatal care and birthweight** | | | | |
| --- | --- | --- | --- | --- |
| **Variable** | **N (%)** |  | **Model 1 ^a^** | **Model 2 ^b^** |
|  |  |  | **β (se)** | |
| **Prenatal care visit frequency** |  |  |  |  |
| Q1(1 ~) | 1828 (27.69) |  | Ref. | Ref. |
| Q2(11 ~) | 2851 (43.19) |  | 27.04 (12.44) ^*^ | 28.38 (12.40) ^*^ |
| Q3(13 ~) | 1078 (16.33) |  | 18.59 (16.40) | 17.10 (16.35) |
| Q4(14 ~) | 844 (12.79) |  | -7.25 (18.17) | -7.86 (18.11) |
| **ANC5** |  |  |  |  |
| Adequate | 5382 (81.53) |  | Ref. | Ref. |
| Inadequate | 1219 (18.47) |  | 2.78 (12.70) | 2.78 (12.70) |
| **APNCU** |  |  |  |  |
| Adequate | 6021 (91.21) |  | Ref. | Ref. |
| Inadequate | 580 (8.79) |  | -69.37 (17.23) ^‡^ | -70.68 (17.18) ^‡^ |
| ^*^ P < 0.05; ^†^ P < 0.01; ^‡^ P < 0.001; ^a^ Model was adjusted with age, education, pre-pregnancy BMI, risk pregnancy, parity, calendar year of the first visit, alcohol or cigarette, and gestational age of the first visit ^b^ Model was adjusted with age, education, BMI, risk pregnancy, parity, calendar year of the first visit, alcohol or cigarette, and gestational age of the first visit. | | | | |

| **Table S9b. Association between prenatal care and LBW and macrosomia** | | | | | | | | | |
| --- | --- | --- | --- | --- | --- | --- | --- | --- | --- |
| **Variable** | **Normal** |  | **LBW** | | |  | **Macrosomia** | | |
|  | **N (%)** |  | **N (%)** | **Model 1 ^a^** | **Model 2 ^b^** |  | **N (%)** | **Model 1 ^a^** | **Model 2 ^b^** |
|  |  |  |  | **OR (95%CI)** | |  |  | **OR (95%CI)** | |
| **Prenatal care visit frequency** |  |  |  |  |  |  |  |  |  |
| Q1(1 ~) | 1588 (26.32) |  | 169 (79.34) | Ref. | Ref. |  | 71 (20.00) | Ref. | Ref. |
| Q2(11 ~) | 2679 (44.41) |  | 34 (15.96) | 0.79 (0.49, 1.28) | 0.79 (0.49, 1.28) |  | 138 (38.87) | 0.97 (0.72, 1.32) | 0.97 (0.72, 1.32) |
| Q3(13 ~) | 1002 (16.61) |  | 7 (3.29) | 1.01 (0.43, 2.38) | 1.01 (0.43, 2.38) |  | 69 (19.44) | 0.96 (0.66, 1.39) | 0.96 (0.66, 1.39) |
| Q4(14 ~) | 764 (12.66) |  | 3 (1.41) | 0.86 (0.25, 2.94) | 0.86 (0.25, 2.94) |  | 77 (21.69) | 1.20 (0.82, 1.75) | 1.20 (0.82, 1.75) |
| **ANC5** |  |  |  |  |  |  |  |  |  |
| Adequate | 4932 (81.75) |  | 171 (80.28) | Ref. | Ref. |  | 279 (78.59) | Ref. | Ref. |
| Inadequate | 1101 (18.25) |  | 42 (19.72) | 1.23 (0.75, 2.02) | 1.23 (0.75, 2.02) |  | 76 (21.41) | 1.23 (0.93, 1.63) | 1.23 (0.93, 1.63) |
| **APNCU** |  |  |  |  |  |  |  |  |  |
| Adequate | 5499 (91.15) |  | 199 (93.43) | Ref. | Ref. |  | 323 (90.99) | Ref. | Ref. |
| Inadequate | 534 (8.85) |  | 14 (6.57) | 1.64 (0.79, 3.40) | 1.63 (0.78, 3.38) |  | 32 (9.01) | 0.73 (0.49, 1.08) | 0.72 (0.49, 1.07) |
| ^*^ P < 0.05; ^†^ P < 0.01; ^‡^ P < 0.001; ^a^ Model was additionally adjusted for age, education, pre-pregnancy BMI, risk pregnancy, parity, calendar year of the first visit, alcohol or cigarette, gestational age of the first visit, and gestational age of delivery ^b^ Model was additionally adjusted for age, education, BMI, risk pregnancy, parity, calendar year of the first visit, alcohol or cigarette, gestational age of the first visit, and gestational age of delivery. | | | | | | | | | |

| **Table S10a. Crossover analysis of ANC5 and APNCU on birth weight** | | | | | |
| --- | --- | --- | --- | --- | --- |
| **ANC5** | **APNCU** | **N (%)** |  | **Model 1 ^a^** | **Model 2 ^b^** |
|  |  |  |  | **β (se)** | |
| Adequate | Adequate | 5165 (78.25) |  | Ref. | Ref. |
| Adequate | Inadequate | 217 (3.29) |  | 38.09 (31.39) | -93.85 (27.06) ‡ |
| Inadequate | Adequate | 856 (12.97) |  | -13.33 (16.72) | 19.16 (14.74) |
| Inadequate | Inadequate | 363 (5.50) |  | 24.99 (24.60) | -49.75 (21.37) * |
| *P*interaction |  |  |  | 0.9958 | 0.4895 |
| ^*^ P < 0.05; ^†^ P < 0.01; ^‡^ P < 0.001; ^a^ Model was adjusted with age, education, pre-pregnancy BMI, risk pregnancy, parity, calendar year of the first visit, alcohol or cigarette, gestational age of the first visit, and gestational age of delivery ^b^ Model was adjusted with age, education, BMI, risk pregnancy, parity, calendar year of the first visit, alcohol or cigarette, gestational age of the first visit, and gestational age of delivery. | | | | | |

| **Table S10b. Crossover analysis of ANC5 and APNCU on LBW and Macrosomia** | | | | | | | | | | |
| --- | --- | --- | --- | --- | --- | --- | --- | --- | --- | --- |
| **ANC5** | **APNCU** | **Normal** |  | **LBW** | | |  | **Macrosomia** | | |
|  |  | **N (%)** |  | **N (%)** | **Model 1 ^a^** | **Model 2 ^b^** |  | **N (%)** | **Model 1 ^a^** | **Model 2 ^b^** |
|  |  |  |  |  | **OR (95%CI)** | |  |  | **OR (95%CI)** | |
| Adequate | Adequate | 4732 (78.44) |  | 166 (77.93) | Ref. | Ref. |  | 267 (75.21) | Ref. | Ref. |
| Adequate | Inadequate | 200 (3.32) |  | 5 (2.35) | 0.71 (0.29, 1.75) | 1.67 (0.51, 5.48) |  | 12 (3.38) | 1.06 (0.59, 1.93) | 0.67 (0.36, 1.25) |
| Inadequate | Adequate | 767 (12.71) |  | 33 (15.49) | 1.23 (0.84, 1.80) | 1.14 (0.66, 1.97) |  | 56 (15.77) | 1.29 (0.96, 1.74) | 1.41 (1.03, 1.94) ^*^ |
| Inadequate | Inadequate | 334 (5.54) |  | 9 (4.23) | 0.77 (0.39, 1.52) | 1.69 (0.69, 4.16) |  | 20 (5.63) | 1.06 (0.66, 1.69) | 0.85 (0.52, 1.38) |
| ***P***_interaction_ |  |  |  |  | 0.8813 | 0.8813 |  |  | 0.7212 | 0.7212 |
| ^*^ P < 0.05; ^†^ P < 0.01; ^‡^ P < 0.001; ^a^  Model was adjusted with age, education, pre-pregnancy BMI, risk pregnancy, parity, calendar year of the first visit, alcohol or cigarette, gestational age of the first visit, and gestational age of delivery; ^b^ Model was adjusted with age, education, BMI, risk pregnancy, parity, calendar year of the first visit, alcohol or cigarette, gestational age of the first visit, and gestational age of delivery. | | | | | | | | | | |

| **Table S11a. Association between prenatal care and PTB** | | | | | | |
| --- | --- | --- | --- | --- | --- | --- |
| **Risk pregnancy** | **Variable** | **PTB** | |  | **Model 1 ^a^** | **Model 2 ^b^** |
|  |  | **No** | **Yes** |  | **OR (95%CI)** | |
|  | **Prenatal care visit frequency** |  |  |  |  |  |
| Low risk | 0 ~ | 8192 (36.16) | 858 (64.85) |  | Ref. | Ref. |
|  | 11 ~ | 3772 (16.65) | 94 (7.11) |  | 0.24 (0.19, 0.29) ^‡^ | 0.22 (0.18, 0.28) ^‡^ |
|  | 12 ~ | 7677 (33.89) | 203 (15.34) |  | 0.25 (0.22, 0.29) ^‡^ | 0.24 (0.20, 0.28) ^‡^ |
|  | 14 ~ | 3013 (13.3) | 168 (12.7) |  | 0.53 (0.45, 0.63) ^‡^ | 0.49 (0.41, 0.58) ^‡^ |
| High risk | 0 ~ | 11535 (34.15) | 1647 (69.91) |  | Ref. | Ref. |
|  | 11 ~ | 6824 (20.2) | 181 (7.68) |  | 0.19 (0.16, 0.22) ^‡^ | 0.18 (0.15, 0.21) ^‡^ |
|  | 12 ~ | 11661 (34.52) | 314 (13.33) |  | 0.19 (0.17, 0.21) ^‡^ | 0.17 (0.15, 0.20) ^‡^ |
|  | 14 ~ | 3761 (11.13) | 214 (9.08) |  | 0.40 (0.34, 0.46) ^‡^ | 0.36 (0.31, 0.41) ^‡^ |
| ***P***_interaction_ | |  | | | 0.0030 | 0.0024 |
|  | **ANC5** |  |  |  |  |  |
| Low risk | Adequate | 15192 (67.06) | 831 (62.81) |  | Ref. | Ref. |
|  | Inadequate | 7462 (32.94) | 492 (37.19) |  | 1.21 (1.07, 1.35) ^†^ | 1.10 (0.96, 1.26) |
| High risk | Adequate | 24214 (71.68) | 1576 (66.89) |  | Ref. | Ref. |
|  | Inadequate | 9567 (28.32) | 780 (33.11) |  | 1.25 (1.15, 1.37) ^‡^ | 1.12 (1.01, 1.24)* |
| ***P***_interaction_ | |  | | | 0.6038 | 0.2969 |
|  | **APNCU** |  |  |  |  |  |
| Low risk | Adequate | 17748 (78.34) | 970 (73.32) |  | Ref. | Ref. |
|  | Inadequate | 4906 (21.66) | 353 (26.68) |  | 1.32 (1.16, 1.49) ^‡^ | 1.23 (1.06, 1.41) ^†^ |
| High risk | Adequate | 28709 (84.99) | 1925 (81.71) |  | Ref. | Ref. |
|  | Inadequate | 5072 (15.01) | 431 (18.29) |  | 1.27 (1.14, 1.41) ^‡^ | 1.14 (1.00, 1.28)* |
| ***P***_interaction_ | |  | | | 0.6535 | 0.9431 |
| ^*^ P < 0.05; ^†^ P < 0.01; ^‡^ P < 0.001; ^a^ Model was crude; ^b^ Model was adjusted with age, education, BMI, parity, calendar year of the first visit, alcohol or cigarette, and gestational age of the first visit. | | | | | | |

| **Table S11b. Association between prenatal care and SGA and LGA** | | | | | | | | | | |
| --- | --- | --- | --- | --- | --- | --- | --- | --- | --- | --- |
| **Risk pregnancy** | **Variable** | **APA** |  | **SGA** | | |  | **LGA** | | |
|  |  | **N (%)** |  | **N (%)** | **Model 1 ^a^** | **Model 2 ^b^** |  | **N (%)** | **Model 1 ^a^** | **Model 2 ^b^** |
|  |  |  |  |  | **OR (95%CI)** | |  |  | **OR (95%CI)** | |
|  | **Prenatal care visit frequency** |  |  |  |  |  |  |  |  |  |
| Low risk | Q1 (0 ~) | 7052 (37.60) |  | 1059 (40.04) | Ref. | Ref. |  | 939 (36.44) | Ref. | Ref. |
|  | Q2 (11 ~) | 2961 (15.79) |  | 470 (17.77) | 1.06 (0.94, 1.19) | 1.04 (0.92, 1.17) |  | 435 (16.88) | 1.10 (0.98, 1.25) | 1.11 (0.98, 1.26) |
|  | Q3 (12 ~) | 6261 (33.38) |  | 804 (30.40) | 0.86 (0.78, 0.94) ^†^ | 0.83 (0.75, 0.92) ^‡^ |  | 815 (31.63) | 0.98 (0.88, 1.08) | 1.00 (0.90, 1.11) |
|  | Q4 (14 ~) | 2481 (13.23) |  | 312 (11.80) | 0.84 (0.73, 0.96) ^†^ | 0.82 (0.71, 0.95) ^†^ |  | 388 (15.06) | 1.17 (1.03, 1.33) ^*^ | 1.16 (1.01, 1.33) ^*^ |
| High risk | Q1 (0 ~) | 10236 (36.32) |  | 1633 (38.13) | Ref. | Ref. |  | 1313 (35.78) | Ref. | Ref. |
|  | Q2 (11 ~) | 5453 (19.35) |  | 831 (19.40) | 0.96 (0.87, 1.05) | 0.92 (0.83, 1.00) |  | 721 (19.65) | 1.03 (0.94, 1.14) | 1.12 (1.01, 1.24) ^*^ |
|  | Q3 (12 ~) | 9435 (33.48) |  | 1356 (31.66) | 0.90 (0.83, 0.97) ^†^ | 0.83 (0.76, 0.90) ^‡^ |  | 1184 (32.26) | 0.98 (0.90, 1.06) | 1.09 (1.00, 1.20) ^*^ |
|  | Q4 (14 ~) | 3060 (10.86) |  | 463 (10.81) | 0.95 (0.85, 1.06) | 0.85 (0.76, 0.96) ^†^ |  | 452 (12.32) | 1.15 (1.03, 1.29) ^*^ | 1.29 (1.14, 1.45) ^‡^ |
| ***P***_interaction_ | |  | | | | 0.3415 |  | | | 0.4063 |
|  | **ANC5** |  |  |  |  |  |  |  |  |  |
| Low risk | Adequate | 12584 (67.10) |  | 1721 (65.07) | Ref. | Ref. |  | 1718 (66.67) | Ref. | Ref. |
|  | Inadequate | 6171 (32.90) |  | 924 (34.93) | 1.09 (1.01, 1.19) ^*^ | 1.10 (0.99, 1.21) |  | 859 (33.33) | 1.02 (0.93, 1.11) | 1.05 (0.95, 1.16) |
| High risk | Adequate | 20234 (71.79) |  | 2977 (69.51) | Ref. | Ref. |  | 2579 (70.27) | Ref. | Ref. |
|  | Inadequate | 7950 (28.21) |  | 1306 (30.49) | 1.12 (1.04, 1.20) ^†^ | 1.16 (1.07, 1.25) ^‡^ |  | 1091 (29.73) | 1.08 (1.00, 1.16) | 1.01 (0.92, 1.10) |
| ***P***_interaction_ | |  | | | | 0.6342 |  | | | 0.5522 |
|  | **APNCU** |  |  |  |  |  |  |  |  |  |
| Low risk | Adequate | 14673 (78.24) |  | 2018 (76.29) | Ref. | Ref. |  | 2027 (78.66) | Ref. | Ref. |
|  | Inadequate | 4082 (21.76) |  | 627 (23.71) | 1.12 (1.01, 1.23) ^*^ | 1.12 (1.01, 1.25) ^*^ |  | 550 (21.34) | 0.98 (0.88, 1.08) | 1.00 (0.89, 1.12) |
| High risk | Adequate | 23961 (85.02) |  | 3597 (83.98) | Ref. | Ref. |  | 3076 (83.81) | Ref. | Ref. |
|  | Inadequate | 4223 (14.98) |  | 686 (16.02) | 1.08 (0.99, 1.18) | 1.10 (1.00, 1.21) |  | 594 (16.19) | 1.10 (1.00, 1.20) | 1.03 (0.93, 1.14) |
| ***P***_interaction_ | |  | | | | 0.1983 |  | | | 0.7539 |
| ^*^ P < 0.05; ^†^ P < 0.01; ^‡^ P < 0.001; Abbreviations: AGA, appropriate for gestaional age; SGA, small for gestional age; LGA, large for gestational age;^a^ Model was crude^b^ Model was additionally adjusted for age, education, BMI, parity, calendar year of the first visit, alcohol or cigarette and gestational age of the first visit. | | | | | | | | | | |

| **Table S12a. Association between prenatal care and birthweight** | | | |
| --- | --- | --- | --- |
| **Risk pregnancy** | **Variable** | **Model 1 ^a^** | **Model 2 ^b^** |
|  |  | **β (se)** | |
|  | **Prenatal care visit frequency** |  |  |
| Low risk | Q1 (0 ~) | Ref. | Ref. |
|  | Q2 (11 ~) | 62.56 (8.38) ^‡^ | 21.55 (7.68) ^†^ |
|  | Q3 (12 ~) | 131.76 (6.72) ^‡^ | 21.77 (6.59) ^‡^ |
|  | Q4 (14 ~) | 198.46 (8.99) ^‡^ | 5.13 (8.94) |
| High risk | Q1 (0 ~) | Ref. | Ref. |
|  | Q2 (11 ~) | 120.98 (6.60) ^‡^ | 40.84 (5.95) ^‡^ |
|  | Q3 (12 ~) | 186.75 (5.63) ^‡^ | 29.30 (5.49) ^‡^ |
|  | Q4 (14 ~) | 246.11 (8.08) ^‡^ | 9.56 (7.81) |
| ***P***_interaction_ | ***P***_interaction_ | < 0.0001 | 0.0117 |
|  | **ANC5** |  |  |
| Low risk | Adequate | Ref. | Ref. |
|  | Inadequate | -4.61 (6.06) | -8.22 (6.07) |
| High risk | Adequate | Ref. | Ref. |
|  | Inadequate | -1.43 (5.30) | -18.08 (5.03) ^‡^ |
| ***P***_interaction_ | ***P***_interaction_ | 0.6944 | 0.1216 |
|  | **APNCU** |  |  |
| Low risk | Adequate | Ref. | Ref. |
|  | Inadequate | 21.64 (6.90) ^†^ | -25.13 (6.74) ^‡^ |
| High risk | Adequate | Ref. | Ref. |
|  | Inadequate | 41.72 (6.67) ^‡^ | -27.35 (6.21) ^‡^ |
| ***P***_interaction_ | ***P***_interaction_ | 0.0370 | 0.8442 |
| ^*^ P < 0.05; ^†^ P < 0.01; ^‡^ P < 0.001; ^a^ Model was crude ^b^ Model was adjusted with age, education, BMI, parity, calendar year of the first visit, alcohol or cigarette, and gestational age of the first visit. | | | |

| **Table S11b. Association between prenatal care and LBW and macrosomia** | | | | | | | | | | | |
| --- | --- | --- | --- | --- | --- | --- | --- | --- | --- | --- | --- |
| **Risk pregnancy** | **Variable** | **Normal** |  | **LBW** | | |  | **Macrosomia** | | | |
|  |  | **N (%)** |  | **N (%)** | **Model 1 ^a^** | **Model 2 ^b^** |  | **N (%)** | **Model 1 ^a^** | **Model 2 ^b^** |  |
|  |  |  |  |  | **OR (95%CI)** | |  |  | **OR (95%CI)** | |  |
|  | **Prenatal care visit frequency** |  |  |  |  |  |  |  |  |  |  |
| Low risk | Q1 (0 ~) | 8162 (37.24) |  | 409 (80.04) | Ref. | Ref. |  | 479 (30.88) | Ref. | Ref. |  |
|  | Q2 (11 ~) | 3606 (16.45) |  | 46 (9.00) | 0.25 (0.19, 0.35) ^‡^ | 0.22 (0.16, 0.30) ^‡^ |  | 214 (13.80) | 1.01 (0.86, 1.19) | 1.06 (0.89, 1.26) |  |
|  | Q3 (12 ~) | 7276 (33.20) |  | 48 (9.39) | 0.13 (0.10, 0.18) ^‡^ | 0.11 (0.08, 0.15) ^‡^ |  | 556 (35.85) | 1.30 (1.15, 1.48) ^‡^ | 1.40 (1.22, 1.60) ^‡^ |  |
|  | Q4 (14 ~) | 2871 (13.10) |  | 8 (1.57) | 0.06 (0.03, 0.11) ^‡^ | 0.04 (0.02, 0.09) ^‡^ |  | 302 (19.47) | 1.79 (1.54, 2.08) ^‡^ | 1.89 (1.60, 2.22) ^‡^ |  |
| High risk | Q1 (0 ~) | 11665 (35.39) |  | 933 (80.85) | Ref. | Ref. |  | 584 (28.84) | Ref. | Ref. |  |
|  | Q2 (11 ~) | 6548 (19.87) |  | 99 (8.58) | 0.19 (0.15, 0.23) ^‡^ | 0.17 (0.14, 0.21) ^‡^ |  | 358 (17.68) | 1.09 (0.96, 1.25) | 1.23 (1.07, 1.41) ^†^ |  |
|  | Q3 (12 ~) | 11142 (33.81) |  | 97 (8.41) | 0.11 (0.09, 0.13) ^‡^ | 0.09 (0.07, 0.11) ^‡^ |  | 736 (36.35) | 1.32 (1.18, 1.48) ^‡^ | 1.52 (1.35, 1.72) ^‡^ |  |
|  | Q4 (14 ~) | 3603 (10.93) |  | 25 (2.17) | 0.09 (0.06, 0.13) ^‡^ | 0.07 (0.05, 0.11) ^‡^ |  | 347 (17.14) | 1.92 (1.68, 2.21) ^‡^ | 2.22 (1.91, 2.58) ^‡^ |  |
| ***P***_interaction_ | |  | | | | 0.4792 |  | | | 0.4306 | |
|  | **ANC5** |  |  |  |  |  |  |  |  |  |  |
| Low risk | Adequate | 14646 (66.83) |  | 334 (65.36) | Ref. | Ref. |  | 1043 (67.25) | Ref. | Ref. |  |
|  | Inadequate | 7269 (33.17) |  | 177 (34.64) | 1.07 (0.89, 1.28) | 1.08 (0.87, 1.33) |  | 508 (32.75) | 0.98 (0.88, 1.10) | 1.01 (0.89, 1.14) |  |
| High risk | Adequate | 23569 (71.51) |  | 808 (70.02) | Ref. | Ref. |  | 1413 (69.78) | Ref. | Ref. |  |
|  | Inadequate | 9389 (28.49) |  | 346 (29.98) | 1.07 (0.95, 1.22) | 1.03 (0.89, 1.19) |  | 612 (30.22) | 1.09 (0.99, 1.20) | 1.02 (0.92, 1.14) |  |
| ***P***_interaction_ | |  | | | | 0.3928 |  | | | 0.7382 | |
|  | **APNCU** |  |  |  |  |  |  |  |  |  |  |
| Low risk | Adequate | 17098 (78.02) |  | 416 (81.41) | Ref. | Ref. |  | 1204 (77.63) | Ref. | Ref. |  |
|  | Inadequate | 4817 (21.98) |  | 95 (18.59) | 0.81 (0.65, 1.02) | 0.76 (0.59, 0.98) ^*^ |  | 347 (22.37) | 1.02 (0.90, 1.16) | 1.07 (0.93, 1.23) |  |
| High risk | Adequate | 27971 (84.87) |  | 1007 (87.26) | Ref. | Ref. |  | 1656 (81.78) | Ref. | Ref. |  |
|  | Inadequate | 4987 (15.13) |  | 147 (12.74) | 0.82 (0.69, 0.98) ^*^ | 0.75 (0.61, 0.91) ^†^ |  | 369 (18.22) | 1.25 (1.11, 1.40) ^‡^ | 1.21 (1.06, 1.37) ^†^ |  |
| ***P***_interaction_ | |  | | | | 0.0697 |  | | | 0.2504 | |
| ^*^ P < 0.05; ^†^ P < 0.01; ^‡^ P < 0.001; ^a^ Model was crude ^b^ Model was additionally adjusted for age, education, BMI, parity, calendar year of the first visit, alcohol or cigarette, gestational age of the first visit, and gestational age of delivery. | | | | | | | | | | | |
